# Susceptibility to allergy in adoptive children: a cross-sectional study at “Bambino Gesù Children’s Hospital”

**DOI:** 10.1186/s13052-017-0440-2

**Published:** 2018-01-04

**Authors:** Hyppolite K. Tchidjou, Maria Fenicia Vescio, Jessica Serafinelli, Rosaria Giampaolo, Alessandro Jenkner, Mathurin C. Tadonkeng, Luca Avellis, Alessandro Fiocchi, Patrizio Pezzotti, Giovanni Rezza, Paolo Rossi

**Affiliations:** 1 Division of Immunology and Infectious Diseases, University-Hospital Pediatric Department (DPUO), Bambino Gesù Children Hospital, Piazza Sant’Onofrio 4, 00165 Rome, Italy; 2 Epidemiology Unit, Department of Infectious Diseases, Istituto Superiore di Sanità, Rome, Italy; 3 Department of Pediatric Medicine, University Department of Pediatrics (DPUO), Children’s Hospital Bambino Gesù, Rome, Italy; 4 Department of Humanistic Studies, Tor Vergata University, Rome, Italy; 5 Division of Allergy, University Department of Pediatrics (DPUO), Children’s Hospital Bambino Gesù, Rome, Italy

**Keywords:** Adopted children, Immigrant children, Allergic state, Child health

## Abstract

**Background:**

Prevalence of allergy has steeply increased during the past few decades, particularly in high-income countries. The development of atopy could present different characteristics in internationally adopted children with regard to incidence, specific patterns of allergies and timing of occurrence. We aimed to investigate the occurrence of allergic diseases among adopted children in Italy.

**Methods:**

We collected demographic information, preadoption immunization data, infectious diseases screening results, immunological status, and performed hematological and biochemical tests according to a standardized protocol in 108 adopted children.

**Results:**

At initial visit (mean age was 5.7 ± 3.2 years), 48 children displayed elevated total serum IgE levels with a prevalence of 56.5% (95%CI: 0.45; 0.67). The prevalences of children screened positive for one or more food allergens and inhalants were 30.1% (95%CI: 19.9%; 42.0%) and 34.3% (95%CI: 23.3%; 46.6%) respectively, only 9 children exhibited abnormal absolute eosinophil counts, 23 (21.3%) had a parasitic infection and 60 (55.6%) had received at least one dose of vaccine.

**Conclusions:**

Children without medical records or with a past medical history suggestive of atopy should perform a thorough allergy evaluation at the time of adoption. Our study offers also a glimpse at the vaccination status and immune-allergic profiles of recent migrant children in Italy.

## Background

Prevalence of allergy is characterized by large geographical variations and has steeply increased during the past few decades, particularly in high-income countries [1–4]. Longitudinal studies on the prevalence of atopy conducted in the US revealed that the prevalence of atopy and asthma varies among racial and ethnic groups, reflecting differences in genetic as well as environmental, social and cultural factors within populations [5, 6]. Nevertheless, how environmental and genetic factors are interrelated and determine the temporal and geographical trends is still poorly understood.

In the last 20 years, we have observed an increase in the prevalence of allergy in Italy, especially among children (from 7 to 25%) [7], making allergy one of the most frequent chronic diseases of the pediatric age in developed countries. This may reflect changes in population composition due to the rising number of immigrants and/or to an increase in the exposure from the non-immigrant population to other additional risk factors (e.g. climate change, bacterial exposure). If on the one hand, migrants are suddenly exposed to a new composition of environment and allergens and as result may develop higher prevalence of allergies, on the other one they should be more protected from allergic diseases compared to non-migrants because of their richer symbiotic microflora [8–10]. Several studies on allergy in immigrant adults indicate [11, 12] that allergy and asthma develop several years after migration to developed countries [13, 14]. Few data are presently available in children [11, 12]. These few data suggest that the development of atopy could present different characteristics in children than in adults both with regard to specific patterns of allergies and to the timing of occurrence [7].

The study of adopted children can provide useful elements for the understanding of atopy in children of recent migration. According to data collected by the Italian Commission for International Adoptions in 2013, Italian families adopted 2825 children coming from abroad, adding up to an approximate number of 33,820 adoptions since 2000. Among Italian regions, the highest number of adoptions was recorded in Lombardy, followed by Latium [15]. If for some aspects, foreign-born adoptees are more easily accessible for a clinical investigation than other groups of recent migrant (legal, illegal) children, this group may differ from the general population of children of recent migration because these are subject to a rapid change of their socio-economic circumstances from low social classes families/orphanages in the country of origin to high social class families in the host country [16].

This study was designed to investigate the occurrence of allergic diseases in adopted children coming from orphanages, their correlates and distinctive features. Building on a previous study in which we have examined children immunization status and their general health [17], this is the first direct investigation on atopy among adopted children in Italy.

## Methods

In Italy, parents of adopted children are advised from the adoption services to attend an International Adoption Unit center for a health checkout of their children in the first few months after adoption. The International Adoption Unit at Bambino Gesù Children’s Hospital, is one of the centers in Rome which provides free access to outpatient care for internationally adopted children. At first visit each child undergoes medical examinations, laboratory screening exams and a psychological interview with the family. Nearly all children attending the center for a first visit are healthy and go there only for a checkup.

From September, 2012 to July, 2016, all the consecutive adopted children referred to the International Adoption Unit at our institution for a first visit were clinically evaluated. At initial medical examination, we collected demographic data, including country of origin and age at adoption. Whenever available, we evaluated health records relating to family history, past and recent medical history, any previous laboratory tests including allergological screening and immunization records (vaccination card from country of origin).

The medical assessment included recollection of any respiratory episodes (wheezing/asthma, sneezing/runny/blocked nose not associated with flu or common cold), food reactions and/or cutaneous symptoms (dermatitis, urticarial rashes, other) possibly related to allergy during the past 12 months. Children were anthropometric evaluated using WHO growth charts for age and sex [18].

Allergy testing included quantitative determination of total serum immunoglobulin E (IgE) levels and specific IgE (sIgE) to food (cow’s milk, Bos d 5, Bos d 6, Bos d 8, egg white, egg yolk, wheat and tomato) and inhalant (dust mites, olive pollen, cynodon dactylon, lolium, perenne, parietaria officinalis, alternaria Tenuis, cat and dog dander) allergens. Patients’ sera from venous blood samples obtained at the time of the first visit were processed with the automated ImmunoCAP system FEIA (Thermo Scientific, Uppsala, Sweden). The cut-off point for positivity was set at 0.1 kUA/L. This assessment was carried out in all children, regardless of their past or present allergy symptoms. We tested specific serum IgE against major food antigens (egg white, egg yolk, milk protein, milk, grain, tomato) and inhalants (dust mite, olive, cynodon, lolium, parietaria, alternaria, cat and dog epithelia).

Hematological and biochemical studies and interferon-gamma release assays were also performed. Immunological status was evaluated by means of serum immunoglobulin (Ig) levels and flow cytometry counts of CD3, CD8 and CD4 lymphocyte subsets. Microbiological tests were also performed in all children in order to identify gastrointestinal parasitic infections.

### Statistical analysis

Age was categorized as less than 2, 3 to 5, and older than 6 years. Descriptive statistical techniques such as median, mean, 95% confidence intervals, interquartile range, frequency and percentages were used to provide a summarized description of the collected information. Country of origin was pooled as continents (Africa, Asia, Europe, and Latin America).

The outcome measures in this analysis were symptoms of allergy (respiratory and cutaneous), total IgE, eosinophil counts and responses to allergy tests (food and inhalant ImmunoCAP specific IgE). Ninety-five percent confidence intervals of the estimated prevalences for these outcomes were calculated using binomial distribution.

Probit regressions with cluster adjusted errors (family level) were carried out to evaluate factors associated with the outcomes, taking into account that some of the adopted children were siblings.

We carried out separate probit regressions for each response and applied the backward stepwise selection procedure to identify predictor variables from those that had a *p*-value <0.20 at the univariate analysis, obtaining a specific set of predictors for each response. Then, we used general non linear Structural Equation Modeling (SEM) [19, 20] to fit a multivariate multiple probit model containing several equations -one for each response- which described the statistical relationship between the retained predictor variables and the responses. Since the responses were assessed on the same set of subjects, and therefore were not independent, we added a random effect at the subject level and constrained the variance of the random effect to 1, to model the correlation between the residuals of the equations. The predicted values are like Z-score. A positive coefficient means that an increase in the predictor leads to an increase in the z-scores. A negative coefficient means that an increase in the predictor leads to a decrease in the z-scores. Only significant effects (from log-likelihood ratio test) were retained. Results were cluster adjusted to take into account the intra sibling correlations. SEM analysis was conducted in STATA 13 [21].

## Results

### Characteristics of the study population

During the study period 108 consecutive, internationally adopted children were enrolled (67 males and 41 females; 37 of them were siblings). The median time between adoption and first visit was 1 month (25q;75q:1; 2 months). Their characteristics are reported in Table 1. Fifty-two children originated from Eastern and Central Europe, 24 from Africa, 15 from Asia and 14 from Latin America. At the time of first clinical evaluation, mean age was 5.7 ± 3.2 years (range: 7 months - 16 years and 7 months). 92 children (85.2%) exhibited poor growth (below the third centile for height and weight), in accordance with WHO growth charts [18] (34/92 of children with poor growth were siblings). One child was above the 97th centile for height (from Ethiopia). A parasitic infection was diagnosed in 23 children (21.3%), 11 (47.8%) of them were from Africa, 9 (39.1%) from Eastern Europe and 3 (13.0%) from Latin America (*p* = 0.062). Regarding serum immunoglobulin levels, two children presented a partial IgA deficiency, three a moderate IgG deficiency (median values 1849 mg/dl; NV 400–1400 mg/dl), and one decreased IgM levels (24 mg/dl; NV 35-210 mg/dl). All these subjects were asymptomatic and had normal lymphocyte subpopulations (CD3^+^, CD4^+^, and CD8^+^). 60 children (55.6%) had received at least one dose of vaccine. 24 out of 47 (51.1%) non-vaccinated children had more than 6 years (*p* = 0.149).

**Table 1:** Respiratory, cutaneous symptoms and selected characteristics of the 108 adoptive children attending the International Adoption Unit at Bambino Gesù Children’s Hospital, Rome, Italy between September 2012 -and July 2016 (variables were not significant at the univariate analysis and therefore were not included in the multivariate analysis)

	ALL		Respiratory symptoms			Cutaneous symptoms		
	N	col%	N	row%	p	N	row%	p
country of origin					0.244			0.457
africa	24	22.22	7	29.17		7	29.17	
latin america	14	12.96	3	23.08		1	7.14	
asia	15	13.89	1	6.67		4	26.67	
eastern europe	52	48.15	11	21.15		12	23.08	
missing	3	2.78	0	0.00		1	33.33	
sex					0.495			0.822
F	41	37.96	7	17.07		9	21.95	
M	67	62.04	15	22.73		16	23.88	
age, years					0.562			0.619
< 3	26	24.07	6	24		7	26.92	
3–5	36	33.33	7	19.44		11	30.56	
6+	45	41.67	8	17.78		7	15.56	
missing	1	0.93	1	100.00		0	0	
poor growth by age & sex					0.836			0.274
no	16	14.81	3	18.75		2	12.5	
yes	92	85.19	19	20.88		23	25	
Stool parasites					0.673			0.369
no	84	77.78	18	21.43		18	21.43	
yes	23	21.3	4	17.39		7	30.43	
missing	1	0.93	0	0		0	0	
vaccination card					0.671			0.417
no	48	44.44	9	18.75		13	27.08	
yes	60	55.56	13	22.03		12	20	

### Signs and symptoms suggestive of allergy

The prevalence of children with respiratory (asthma, recurrent respiratory infection, wheezing, bronchospasm, and rhinitis) or cutaneous (dermatitis or urticarial rash) signs and symptoms suggestive of allergy was 20.6% (95%CI: 13.4%; 29.5%) and 23.1% (95%CI: 15.6%; 32.2%) respectively (see Table 1). 2 children presented both cutaneous and respiratory symptoms (both were from Africa, had no evidence of concurrent parasitic disease and exhibited normal eosinophil counts and IgE levels). None of the children had chronic gastrointestinal symptoms that could pose a clinical suspicion of allergy and none of the children had otitis. There were no specific characteristics that resulted to be significantly associated with these signs or symptoms.

### Total serum IgE levels and absolute eosinophil counts

Total serum IgE levels exceeded age standardized normal values in 48 children with a prevalence of 56.5% (95%CI: 0.45; 0.67). Prevalence ranged between 74% for children of African origin and 45% for those from Latin America (*p* = 0.159) and reached 71.4% (95%CI: 47.8%; 88.7%) in children with intestinal parasites (*p* = 0.183). 9 (2.9%; 95%CI: 0.6%; 8.1%) children had absolute hypereosinophilia (8 males and 1 female, *p* = 0.103) and 8 of them (17.4%, *p* = 0.034) had also high total IgE levels (multivariate probit model rho: 0.55; 95%CI: 0.17; 0.93). (see Table 2). The association between eosinophil counts and sex remained significant at the multivariate analysis (coef. 0.64; 95%CI: -0.19; 1.46; *p* = 0.131).

**Table 2:** IgE, EO and selected characteristics of the 108 adoptive children attending the International Adoption Unit at Bambino Gesù Children’s Hospital, Rome, Italy between September 2012 and July 2016 - estimated Z-scores from the multivariate probit model (NI: variable not included in the multivariate analysis because - not significant at the univariate analysis, −excluded by stepwise algorithm, 95%CI: 95% confidence intervals)

	ALL		Total IgE levels						Eosinophil counts					
	N	col%	N	row%	p	coef	(95%CI)	p	N	row%	p	coef	(95%CI)	p
country of origin					0.159	NI					0.370	NI		
africa	24	22.22	14	73.68					3	13.64				
latin america	14	12.96	5	45.45					1	7.69				
asia	15	13.89	5	50.00					1	6.67				
eastern europe	52	48.15	22	52.38					4	7.69				
missing	3	2.78	2	66.67					0	0.00				
sex					0.764	NI					0.103			
F	41	37.96	18	54.55					1	2.56		ref		
M	67	62.04	30	57.69					8	12.12		0.64	(−0.19; 1.46)	0.131
age, years					0.249	NI					0.312	NI		
< 3	26	24.07	8	44.44					1	3.85				
3–5	36	33.33	17	60.71					4	11.76				
6+	45	41.67	23	60.53					4	9.09				
missing	1	0.93	0	0.00					0	0.00				
poor growth by age & sex					0.959	NI					0.558	NI		
no	16	14.81	8	57.14					2	12.5				
yes	92	85.19	40	56.34					7	7.87				
stool parasites					0.183						0.367	NI		
no	84	77.78	33	51.56		ref			6	7.32				
yes	23	21.3	15	71.43		0.52	(−0.33; 1.37)	0.228	3	13.64				
missing	1	0.93	0	0.00					0	0.00				
vaccination card					0.449	NI					0.488	NI		
no	48	44.44	23	62.16					5	10.64				
yes	60	55.56	25	52.08					4	6.90				

### Food and inhalant specific tests

Food and inhalant specific test results are reported in Table 3 (see Table 4 for a list of the most frequent allergens observed in this population). 22 out of 73 (30.1%; 95%CI: 19.9%; 42.0%) children resulted positive for one or more food allergens. 7 (31.8%) children had respiratory symptoms, 9 (40.9%) presented cutaneous manifestations, 5 (22.7%, *p* = 0.097) displayed absolute hypereosinophilia and 16 had elevated total IgE levels (72.7%, *p* = 0.070). 35 children did not perform food tests. 24 out of 70 (34.3%: 95%CI: 23.3%; 46.6%) children tested positive for one or more inhalants (38 children did not perform inhalant tests): 5(20.8%) had respiratory symptoms and 8(33.3%) cutaneous manifestations, 4(17.4%, *p* = 0.479) displayed absolute hypereosinophilia and 19(79.2%, *p* = 0.010) had elevated total IgE counts. 11 (16.4%) children were positive to both food and inhalant allergens and 4 of them had parasitic infections and high total serum IgE levels. 5(10.4%) children with high IgE levels had negative sIgE and no allergic manifestations. Children positive to inhalants sIgE tended to be older than their negative counterparts (*p* = 0.072). These results were confirmed in the multivariate analysis that showed that children aged between 3 and 5 years had a higher risk of being positive for respiratory allergens (0.67; 95%CI: -0.29; 1.63; *p* = 0.170) than children younger than 3 or older than 5 years. At the multivariate analysis total IgE levels were significantly correlated with inhalant sIgE (rho: 0.48; 95%CI: 0.05; 0.91).

**Table 3:** Food, inhalant immunoCAP specific IgE and selected characteristics of the 108 adoptive children attending the International Adoption Unit at Bambino Gesù Children’s Hospital, Rome, Italy between September 2012 -and July 2016- estimated Z-scores from the multivariate probit model (NI: variable not included in the multivariate analysis because - not significant at the univariate analysis, −excluded by stepwise algorithm; sIgE: specific IgE, 95%CI: 95% confidence intervals)

	ALL		ImmunoCAP inhalant sIgE						ImmunoCAP food sIgE					
	N	col%	N	row%	p	coef	(95% CI)	p	N	row%	p	coef	(95%CI)	p
country of origin					0.755	NI					0.856	NI		
africa	24	22.22	6	37.5					5	29.41				
latin america	14	12.96	4	50					2	25				
asia	15	13.89	1	14.29					3	33.33				
eastern europe	52	48.15	12	33.33					12	33.33				
missing	3	2.78	1	33.33					0	0.00				
sex					0.717	NI					0.715	NI		
F	41	37.96	10	37.04					8	27.59				
M	67	62.04	14	32.56					14	31.82				
age, years					0.072						0.948	NI		
< 3	26	24.07	2	14.29		ref			5	31.25				
3–5	36	33.33	10	43.48		0.67	(−0.29; 1.63)	0.170	10	38.46				
6+	45	41.67	12	37.50		0.62	(−0.38; 1.43)	0.253	7	23.33				
missing	1	0.93	0	0.00					0	0.00				
poor growth by age & sex					0.356	NI					0.507	NI		
no	16	14.81	3	23.08					3	23.08				
yes	92	85.19	21	36.84					19	31.67				
stool parasites					0.192	NI					0.987	NI		
no	84	77.78	15	29.41					16	30.19				
yes	23	21.3	9	47.37					6	30.00				
missing	1	0.93	0	0.00					0	0.00				
vaccination card					0.631	NI					0.851	NI		
no	48	44.44	10	31.25					10	31.25				
yes	60	55.56	14	36.84					12	29.27

**Table 4:** List of food and inhalants sIgE found in the 108 adoptive children attending the International Adoption Unit at Bambino Gesù Children’s Hospital, Rome, Italy between September 2012 -and July 2016

	N	col%
food antigens		
milk	15	68.18
egg	9	40.91
wheat	3	13.64
tomato	3	13.64
nut	1	4.55
inhalants		
cynodon	4	16.67
dermathophagoides	18	75.00
cat	2	8.33
olea	3	12.50
parietaria	5	20.83
alternaria	5	20.83
lolium	3	12.50

## Discussion

International adoption involves not only exposure to a new macroenvironment, but it also implies significant socioeconomic and cultural changes, such as housing, diet and access to medical services [11]. A history of admission to unsuitable environments (orphanage, shelter, children’s home or other institution) at a young age, bringing about lack of stimulation, nutritional deficiencies and poor hygiene may influence the physical and mental health of children, making them individuals requiring special care [1]. Appropriate and specific screening tests are required in this population in addition to a standard well-child visit, especially to reduce the burden on the national health system in the period following the adoption [12, 22–24].

A few studies have evaluated allergy-prevalence among internationally adopted children [9, 12]. To the best of our knowledge, our study is the first providing specific data on the susceptibility of atopy in adoptive children in Italy. We studied 108 previously institutionalized, internationally adopted children. Such analysis concentrated on investigating factors that might have contributed to allergy (clinical history, parasitic co-infection and previous immunizations). Adopted children came from various countries many of which with lower prevalence of atopy than Italy (wheezing: Albania, Romania, India, Morocco and China, atopic eczema: Albania, China and India) [4, 25]. At initial visit, 20.6% of children presented with an history of respiratory symptoms attributable to allergy, while 23.1% had cutaneous clinical manifestations of allergy and between 30.1% to 34.3% tested positive to food or inhalants sIgE. These proportions were higher than those observed for children aged 9–13 years in Italy (food allergy prevalences range between 4 and 11%) [12, 24]. Our estimates were also different from those observed for migrant children living in Italy [25, 26] and other western countries [27]. The SIDRIA 2 study, a large multi-center cross-sectional study of children attending the first 2 years of primary school and the last year of secondary school in northern, center and southern Italy, for example, found that in first 2 years of life respiratory symptoms suggestive of allergy were more likely to occur in children born in Italy than in migrants and, that among the latter, the prevalence of allergic symptoms reflected those of the region of origin (e.g. relatively lower prevalences for current wheezing were found for children born in eastern Europe, Africa and in Asia and higher ones for children born in South America) [25]. In the same way, another study carried out in Mantua Northern Italy (Viadana study), found that the sex/age-adjusted incidence rates of wheezing and eczema were the highest for children born in Italy to Italian parents, tended to decrease for those born to foreign parents and were the lowest for children born abroad [26]. The only exception was found for persistent cough or phlegm which resulted prevalent in children born in Italy from foreign parents than in those born to Italian parents. Furthermore, a recent multicenter cross-sectional study carried out in Italy which compared children born in Italy from Italian parents and children born either in Italy or abroad from immigrants, found that there were no clinical differences between immigrants and Italian children with regard to the severity of respiratory allergy or the presence of a history of food allergy or atopic dermatitis. However this study included children referred for allergic respiratory diseases (rhinitis/asthma), with an ascertained clinical diagnosis and IgE sensitization to inhalants and did not take into account children adoptive status and their socio-economic circumstances. In addition immigrant children were slightly older than Italians [12, 24–26].

Foreign-born adoptees differ in several aspects from other immigrants (e.g. adoptees come from orphanage, are adopted by high social class parents and are rapidly integrated in the host country lifestyle). An article which analyzed data from the Swedish Prescribed Drug Register, found that the purchase of prescribed inhaled corticosteroids (which was used as a marker of asthma) during 2006 was more likely in adoptees from Asia and Latin America when compared with Swedish-born subjects with Swedish-born parents and less likely in adoptee from Eastern Europe [16]. Purchases were instead lower for immigrant children and for children born in Sweden to foreign parents [16]. However, data for inhaled cortisone were collected years after adoption, and estimates adjusted by age and sex of the child but not by time since adoption.

Children positive to inhalants were older than their counterparts with negative test results, although results achieved significance only for children aged 3–5 years at the multivariate analyses but not for those above the age of 5. Conversely, among immigrant children living in Sweden the highest risk of asthma was found in children adopted in the first 2 years of life and was reduced progressively with age at adoption reaching the lowest values for those who had been adopted after 5 years of age [16].

Estimates of atopy in our populations did not vary significantly by country of origin. However, due to the low numbers we cannot exclude that some groups (e.g. children of African origin) were more susceptible to atopy than others when exposed to the environment of a high-income country. Since medical examination and sIgE were carried out within few weeks after the children arrival in Italy these reflect the sensitization adoptee arrived with and not that developed because of the exposure to a new environment. If for adopted children prevalence of atopy increases as for other immigrants with the length of stay in the host country, we may expect in the future an increase in the estimates of atopy in this population.

We found that 56.5% of children exceeded normal age-adapted values for IgE and 31.3% of them were diagnosed with a parasitic infection. As numerous outbreaks in institutionalized settings have been reported, it is not surprising that intestinal protozoa are frequently identified on routine screening of international adoptees [11, 28–30]. In a 2005 study, Murray identified that intestinal parasites are common in international adoptees children, with a prevalence of 14% to 33%. The most common intestinal parasite was Giardia lamblia; these studies also identified a substantial number of individuals with more than one parasite [28]. The immunologic mechanisms responsible for IgE production that are protective in helminthiasis parasites infections are similar to those involved in the production of specific IgE against allergens. Therefore, helminths may decrease the risk of allergies by stimulating the production of high levels of polyclonal IgE that are capable of blocking Fragment Crystallizable (Fc) receptors on mast cells, or by promoting high levels of regulatory cytokines capable of downregulating the allergic response [31].

Moreover, elevated total IgE levels have been associated with an increased risk of allergic diseases [32] and can antedate the onset of symptoms by several years [33, 34]. Nevertheless, the use of total IgE as an allergy screening test is scarce due to a lack of accuracy [35].

It is remarkable that only the 55.6% of children had received at least one dose of vaccine and that the 51.1% of the not vaccinated were older than 6 years as also observed in our previous study [17].

Some limitations of our study should be taken into account, including small sample size (in face of great patient heterogeneity which is partly due to the different prevalence of atopy in the countries of origin of children), lack of information regarding exposure to environmental factors and genetic predisposition (incomplete family history, HLA diagnosis), and the paucity of past medical documentation. In addition, children were enrolled on a voluntary basis. Selection bias might have occurred if non participants were any different from those included in the study with regard to clinical manifestations of atopy and/or positivity to sIgE and/or factors to them correlated to some extent. Perhaps, our estimates may be overestimated if symptomatic children had been more likely to attend the center than non-symptomatic ones. However, this seems unlikely to have occurred since the Bambino Gesù Children’s Hospital is a reference center for international adoptive children in Rome and offers evaluation screenings free of charge. It is not inconceivable that the time between the adoption and the first visit may vary depending on the clinical condition of the child. Given that allergy symptoms increase with time spent in the host country, if indeed asymptomatic children were brought to the center later than those with symptoms, it would follow an over estimation of the prevalence of allergy in our population. However, nearly all the children afferent to the center were healthy and were visited within the first 3 months from adoption. About 30% of children (32% did not perform food tests and 35% inhalant tests) did not undergo allergologic tests and results of these tests could be over-estimated if the not tested were less likely to result positive than those tested.

In addition, the tested allergen panel was not complete and in particular did not contain allergens typical of the children’s country of origin. Therefore susceptibility to any allergen not tested in this study was not detected.

On the other hand, our study has several strengths. Differently from other studies which used self-reported information, each child was visited by a doctor. Signs and symptoms suggestive of allergy were investigated thoroughly, to make differential diagnosis with other diseases (e.g. respiratory flu symptoms). Total serum IgE levels, eosinophil counts, tests for the detection of parasites were carried out during the first visit in all children regardless of the presence or absence of symptoms suggestive of atopy which has allowed us to also identify cases with few symptoms.

## Conclusion

In conclusion, our study emphasizes the importance both of careful clinical examination and of laboratory screening after adoption, and offers a glimpse at the infectious diseases, vaccination status and immune-allergic profiles of migrant children at their arrival in Italy, including the non adopted ones, which are more difficult to investigate. The need of performing a cost/benefit allergy screening in children with unavailable medical records or with a medical history suggestive of allergic or parasitic diseases is underestimated. The clinical management of children with a suspicion of allergy should also take into account their adoptive status.
